# A novel ethanol gas sensor based on TiO_2_/Ag_0.35_V_2_O_5_ branched nanoheterostructures

**DOI:** 10.1038/srep33092

**Published:** 2016-09-12

**Authors:** Yuan Wang, Lixin Liu, Chuanmin Meng, Yun Zhou, Zhao Gao, Xuhai Li, Xiuxia Cao, Liang Xu, Wenjun Zhu

**Affiliations:** 1 National Key Laboratory of Shock Wave and Detonation Physics, Institute of Fluid Physics, China Academy of Engineering Physics, P. O. Box 919-111, Mianyang, Sichuan 621900, People’s Republic of China

## Abstract

Much greater surface-to-volume ratio of hierarchical nanostructures renders them attract considerable interest as prototypical gas sensors. In this work, a novel resistive gas sensor based on TiO_2_/Ag_0.35_V_2_O_5_ branched nanoheterostructures is fabricated by a facile one-step synthetic process and the ethanol sensing performance of this device is characterized systematically, which shows faster response/recovery behavior, better selectivity, and higher sensitivity of about 9 times as compared to the pure TiO_2_ nanofibers. The enhanced sensitivity of the TiO_2_/Ag_0.35_V_2_O_5_ branched nanoheterostructures should be attributed to the extraordinary branched hierarchical structures and TiO_2_/Ag_0.35_V_2_O_5_ heterojunctions, which can eventually result in an obvious change of resistance upon ethanol exposure. This study not only indicates the gas sensing mechanism for performance enhancement of branched nanoheterostructures, but also proposes a rational approach to design nanostructure based chemical sensors with desirable performance.

Nowadays, atmospheric pollution has become a critical problem for modern society, In order to control such emission, gas sensors for the quantitative detection of different toxic gases have been widely developed due to their low power, high response, prominent selectivity, good repeatability and stability^1^. Till now, various kinds of gas sensors, including metal oxide gas sensors^2^,^3^,^4^, solid electrolyte gas sensors^5^, electrochemical gas sensors^6^, graphene-based gas sensors^7^, and organic compounds gas sensors^1^, have been extensively explored. Among these different gas sensors, resistance-type metal oxide semiconductor gas sensors have attracted great attention in the past few years since their high sensitivity to most gases, low cost, and simple fabrication techniques^8^,^9^. Since the first demonstration of gas sensors based on metal oxide by Seiyama^10^, an enormous amount of effort has been invested in investigating the sensing performance of metal oxide based sensors^11^,^12^,^13^.

As a representational metal oxide semiconductor, TiO_2_ has been extensively studied and considered as one of the most promising materials for gas detection due to its high chemical and mechanical stabilities, harsh environment tolerance, environmentally friendly characters and catalytic properties^14^. Unfortunately, as a high resistance n-type semiconductor, the widely application of TiO_2_-based gas sensors is influenced by its low sensitivity, long response and recovery time, and high working temperature. Recently, a great many effort has been developed to enhance the gas sensing performance of TiO_2_, such as nanostructured materials with ultra-high surface-to-volume ratios^15^. element doping^16^, surface modification with noble metals^17^, semiconductor-semiconductor heterostructural nanomaterials, and so on. Remarkably, TiO_2_-based two-component heterostructures, such as TiO_2_/SnO_2_^18^, TiO_2_/ZnO^19^,^20^ and TiO_2_/Fe_2_O_3_^21^,^22^ with improved sensing properties have been successfully prepared. Referring the band matching and gas sensing mechanism of the heterostructures, it can be obtained that the semiconductors for TiO_2_ incorporation should possess appropriate energy levels to form apposite energy barrier at the heterojunction interface (the band gap, work function and electron affinity of TiO_2_ are 3.2, 4.2, and 3.9 eV, while that of Fe_2_O_3_ are 2.1, 5.6, and 4.71 eV, and that of ZnO are 3.2, 5.2, and 3.9 eV, respectively). As is well known, energy barrier height can be adjusted in different gas condition, thus sensing performance of the heterostructure can be enhanced by coupling two semiconductors with matched energy levels to make heterostructure act as a lever in electron transfer which can be facilitated or restrained through the change of energy barrier height. This could be suitable not only for TiO_2_, but also for other metal oxide based gas sensors^23^. Therefore, establishing heterostructures in sensor materials has long been regarded as the best strategy.

Recently, Silver vanadium oxides, such as AgVO_3_ and Ag_0.35_V_2_O_5_, have attracted increasing attention for their application in batteries because of their unique electronic structure^24^. In particular, it has been reported that the electrical conductivity of Ag_0.35_V_2_O_5_ nanowires is 0.5 S/cm, about 6–7 times higher than that of V_2_O_5_ nanowires^25^, and the amine sensitivity of Ag_0.35_V_2_O_5_ is much higher compared with V_2_O_5_ particles^26^. Accordingly, it may be an interesting role to modify TiO_2_ with Ag_0.35_V_2_O_5_ to get enhanced gas sensitivity. However, to the best of our knowledge, there has been no report so far on the gas sensing performance of TiO_2_/Ag_0.35_V_2_O_5_ composite. Furthermore, the emergence of nanostructures, such as one-dimensional (1D) nanomaterials (nanowires, nanorods, nanofibers), have led to improved sensitivity compared with conventional thin film due to their largely increased surface to volume ratio and rich surface chemistry on the nanostructure surfaces^27^.

Accordingly, in this paper, a novel ethanol gas sensor based on TiO_2_/Ag_0.35_V_2_O_5_ nanoheterostructures with branched fiber-structures prepared by a facile one-step synthetic process is presented, in which well-matched energy levels are induced by the formation of effective heterojunctions between TiO_2_ and Ag_0.35_V_2_O_5_, and at the same time, the branched-nanofiber structures display large Brunauer-Emmett-Teller (BET) surface area and complete electrons depletion for the nanobranches. By this way, the TiO_2_/Ag_0.35_V_2_O_5_ branched nanoheterostructures sensor exhibits higher selectivity, shorter response and recovery time, and higher sensitivity than pure TiO_2_ nanofibers.

## Results and Discussion

### Structure and morphology

**Figure 1:**
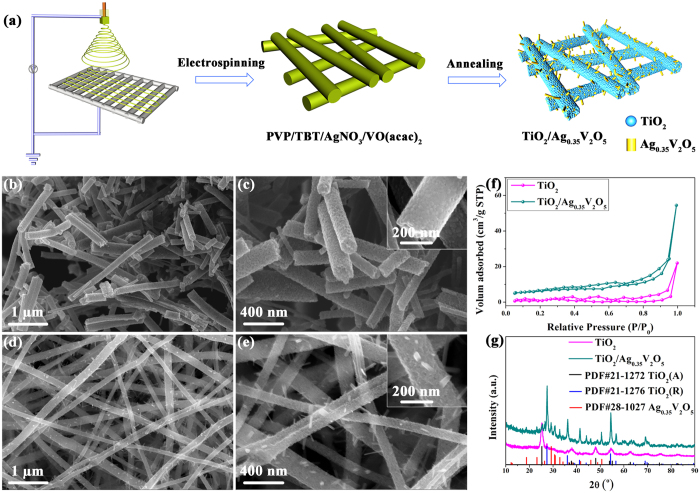
Fabrication of TiO_2_/Ag_0.35_V_2_O_5_ branched nanoheterostructures and characters of the heterostructures. (**a**) Schematic illustration of the fabrication process for TiO_2_/Ag_0.35_V_2_O_5_ branched nanoheterostructures, which are first prepared by electrospinning and then annealed at 450 °C in ambient air. (**b**–**e**) SEM images at three different magnifications of TiO_2_ nanofibers (**b**,**c**) and TiO_2_/Ag_0.35_V_2_O_5_ branched nanoheterostructures (**d**,**e**), where a great many small branches extend out of the fiber backbones. (**f**) N_2_ adsorption–desorption isotherms and (**g**) XRD patterns of TiO_2_ nanofibers and TiO_2_/Ag_0.35_V_2_O_5_ branched nanoheterostructures.

The TiO_2_/Ag_0.35_V_2_O_5_ branched nanoheterostructures are composed of two phases: crystalline TiO_2_ as the host, Ag_0.35_V_2_O_5_ is introduced as the activators (right hand side of Fig. 1a). The process for fabricating the TiO_2_/Ag_0.35_V_2_O_5_ heterostructures is based on a one-step electrospinning approach (Fig. 1a). Briefly, continuous PVP/tetrabutyl titanate/silver nitrate/vanadyl acetylacetonate (PVP/TBT/AgNO_3_/VO(acac)_2_) nanofibers are prepared by means of electrospinning, and then the nanofibers are annealed in air ambient to crystallize the oxides and remove the PVP support (See the methods for details).

The microstructures of the samples are investigated by SEM images. As shown in Fig. 1b,c, pure TiO_2_ nanofibers with rough surface and uniform morphology can be observed, diameter of the nanofibers is approximately 220 nm and the length is about several micrometers. After introducing Ag_0.35_V_2_O_5_, nanofibers become thoroughly rougher and a great many nanobranches owing to the secondary growth of Ag_0.35_V_2_O_5_ distribute uniformly on the surface of them, where diameter of the nanofibers is about 190 nm and that of nanobranches is about 20 nm (as shown in Fig. 1d,e). These novel branched nanostructures can provide more active sites for absorption of gas molecular and reaction of gas molecular with surface-adsorbed oxygen ions, thus would be benefit to the gas sensing response.

For a good understanding of the influence of the nanoheterostructure on the gas sensing performance, we use BET method of adsorption and desorption of nitrogen gas to measure the specific surface area of the TiO_2_/Ag_0.35_V_2_O_5_ branched nanoheterostructures and pure TiO_2_ nanofibers, as shown in Fig. 1f. The BET surface area of TiO_2_/Ag_0.35_V_2_O_5_ branched nanoheterostructures calculated from the nitrogen isotherm is 21.15 m^2^g^−1^, of about five times that of pure TiO_2_ nanofibers (4.78 m^2^g^−1^). Obviously, the enhanced surface area of the TiO_2_/Ag_0.35_V_2_O_5_ branched nanoheterostructures is mainly attributed to the growth of nanobranches on the nanofibers surface.

XRD patterns have been employed to identify the phase composition and crystal structure of the samples (Fig. 1g). It can be seen that all the samples exhibit strong diffraction peaks, demonstrating the high crystallinity of the samples. The diffraction peaks of the pure TiO_2_ nanofibers match the standard patterns of the rutile and anatase phase TiO_2_ (PDF#21-1276, PDF#21-1272). As for the TiO_2_/Ag_0.35_V_2_O_5_ branched nanoheterostructures, several additional diffraction peaks can be clearly observed compared with the pure TiO_2_ nanofibers, which can be indexed to the diffraction pattern of monoclinic Ag_0.35_V_2_O_5_ (PDF#28-1027), indicating the TiO_2_/Ag_0.35_V_2_O_5_ branched nanoheterostructures composed of anatase TiO_2_, rutile TiO_2_, and monoclinic Ag_0.35_V_2_O_5_ have been successfully prepared by the one-step electrospinning process. Moreover, the color of the two samples is very different, as can be clearly seen in Fig. S1, the color of TiO_2_ nanofibers is white, while the TiO_2_/Ag_0.35_V_2_O_5_ branched nanoheterostructures turn to brown, indicating Ag_0.35_V_2_O_5_ are successfully introduced to TiO_2_ host, this can also be confirmed by the enhanced visible light absorption of the TiO_2_/Ag_0.35_V_2_O_5_ branched nanoheterostructures compared with the pure TiO_2_ nanofibers (Fig. S2a). Additionally, the incorporation of Ag_0.35_V_2_O_5_ leads to an increase of the phase transition of TiO_2_ from anatase to rutile, this effect can also be observed in other TiO_2_ based materials, such as TiO_2_/V_2_O_5_^28^.

**Figure 2:**
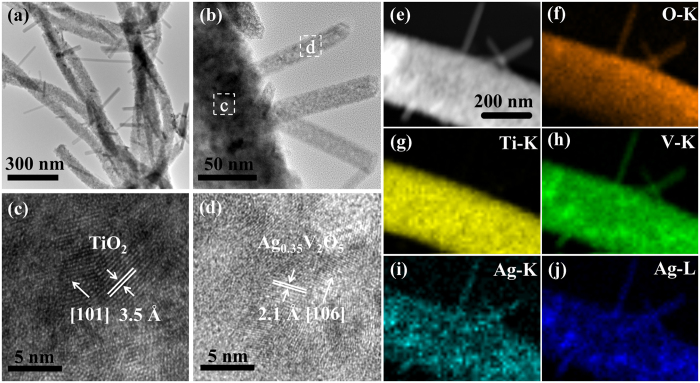
Morphology and structure of TiO_2_/Ag_0.35_V_2_O_5_ branched nanoheterostructures. (**a**) TEM image of the nanoheterostructures, illustrating the formation of branched fiber-like nanostructures. (**b**) High-magnification TEM (HRTEM) image of the TiO_2_/Ag_0.35_V_2_O_5_ branched nanoheterostructure. (**c**,**d**) Magnified parts of the typical backbone and branch taken from the boxed areas in (**b**). (**e**–**j**) STEM image of the TiO_2_/Ag_0.35_V_2_O_5_ branched nanoheterostructure (**e**) and corresponding EDX elemental maps of O (**f**) Ti (**g**) V (**h**) and Ag (**i**,**j**), respectively, where only O, V, and Ag elements present in the branches.

To further study the microscopic morphology and structure information of the as-synthesized TiO_2_/Ag_0.35_V_2_O_5_ branched nanoheterostructures, TEM analysis is performed, as shown in Fig. 2. Branched-fiber-like structure of the TiO_2_/Ag_0.35_V_2_O_5_ nanoheterostructures is clearly evidenced in Fig. 2a,b, where nanobranches of 10–20 nm in diameter are well dispersed on the surface of the nanofibers. HRTEM images of the backbone and branch defined by white boxes in Fig. 2b are shown in Fig. 2c,d, respectively. It can be seen that a strong alignment of two different crystal lattices resulted from the epitaxial growth of Ag_0.35_V_2_O_5_ on TiO_2_ is displayed obviously. The measured lattice distance of 3.5 Å corresponds to the (101) lattice distance of anatase TiO_2_, and the lattice fringe of 2.1 Å corresponds to the interplanar spacing of (106) planes of monoclinic Ag_0.35_V_2_O_5_. In addition, in order to further identify the elements distribution of the nanoheterostructures, STEM-EDS elemental mapping analysis is employed, as can be seen clearly from Fig. 2e–j, the nanofiber is mainly composed of O, Ti, V, and Ag elements, whereas the nanobranches only consist of O, V, and Ag elements, indicating the nanobranches are made of Ag_0.35_V_2_O_5_ and the parent nanofibers are still a mixture of TiO_2_ and Ag_0.35_V_2_O_5_. The microstructures in Fig. 2 imply that the secondary growth of Ag_0.35_V_2_O_5_ does appear here, consistent with the SEM results.

**Figure 3:**
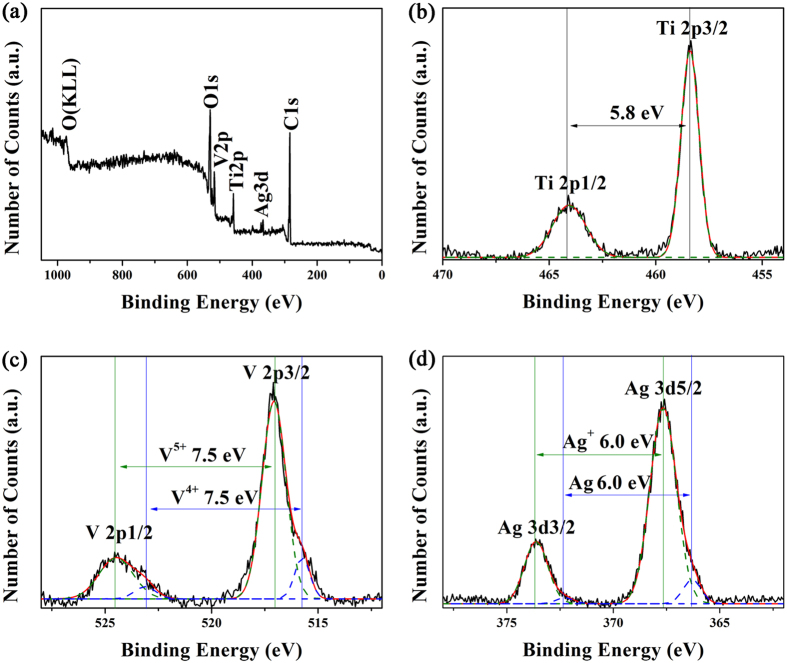
XPS spectra of TiO_2_/Ag_0.35_V_2_O_5_ branched nanoheterostructures. (**a**) survey spectrum, (**b**–**d**) high resolution XPS spectra of Ti 2p, V 2P, and O 1s core-level binding energy, respectively.

To determine the chemical composition of the nanoheterostructures, XPS measurements are carried out in the region of 0–1050 eV (Fig. 3 and Fig. S3), in which all binding energies are calibrated to the C 1s peak at 284.6 eV (Fig. S3b). The whole survey for all elements detection of the TiO_2_/Ag_0.35_V_2_O_5_ branched nanoheterostructures is presented in Fig. 3a, where O, V, Ti, Ag and C are detected. For comparison, the XPS whole survey of pure TiO_2_ nanofibers is displayed in Fig. S3a, where only O, Ti, and C are detected. The two well resolved peaks at 458.6 and 464.2 eV observed from the Ti 2p core-level spectrum (Fig. 3b) can be ascribed to the Ti 2p3/2 and Ti 2p1/2 spin-orbital components, respectively, which are characteristic of a +4 oxidation state of titanium^29^. The V 2p core-level spectrum of the TiO_2_/Ag_0.35_V_2_O_5_ branched nanoheterostructures is shown in Fig. 3c, the V 2p3/2 and V 2p1/2 peaks located at 517.1 and 524.6 eV is consistent with a +5 oxidation state of the vanadium^30^. In addition, two small peaks at 515.8 eV and 523.0 eV indicate the appearance of V^4+^ during the preparation process^30^. It is calculated that the molar ratio of V^4+^ to V^5+^ is 0.13. Fig. 3d shows that the silver species in the TiO_2_/Ag_0.35_V_2_O_5_ sample include Ag^+^ and metallic Ag^25^. The metallic Ag is not explored in XRD pattern may be because the little quantity and the no organization in a long range order. The atomic ratio of metallic Ag to Ag^+^ in the TiO_2_/Ag_0.35_V_2_O_5_ branched nanoheterostructures is calculated to be 0.08, and thus the chemical composition of Ag_0.35_V_2_O_5_ should be Ag_0.026_Ag^+^_0.324_V^4+^_0.23_V^5+^_1.77_O_5_.

### Gas sensing properties

The resistance of the sensor is measured under the conditions of exposing the TiO_2_/Ag_0.35_V_2_O_5_ branched nanoheterostructures based sensor to ethanol vapor and dry air alternately. Sensor response to the gas is expressed with the normalized value R_a_/R_g_, where R_a_ is the initial value in air and R_g_ is the initial value in ethanol vapor exposure. In addition, the sensor’s repeatability and sensor drift are studied by subsequent exposure-cleaning cycles. Due to good work function matching, the role of the contact between the semiconducting TiO_2_/Ag_0.35_V_2_O_5_ and the gold electrodes seems to have a negligible effect on the conduction.

**Figure 4:**
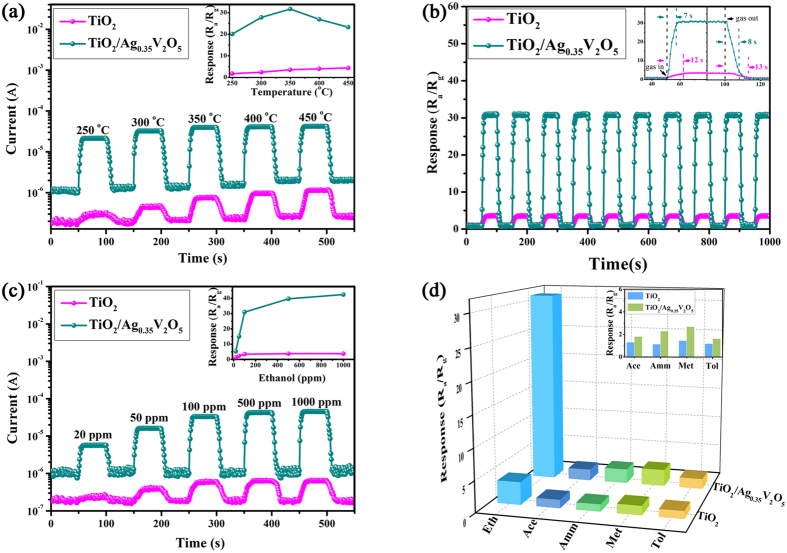
Gas sensing performance of the sensors. (**a**) Gas sensing properties versus different operating temperatures of the pure TiO_2_ nanofibers and TiO_2_/Ag_0.35_V_2_O_5_ branched nanoheterostructures based sensors exposed to 100 ppm ethanol. The inset shows the corresponding responses. (**b**) Reproducibility of the two sensors exposed to 100 ppm successive ethanol vapors (10 cycles) at 350 °C. The inset is the typical response and recovery curves of the two different types of sensors. (**c**) Dynamic response-recovery curves of the two sensors to ethanol vapors at 350 °C in the concentration sequence of 20, 50, 100, 500 and 1000 ppm. The inset is the corresponding responses. (**d**) Selective tests of TiO_2_/Ag_0.35_V_2_O_5_ branched nanoheterostructures compared with TiO_2_ nanofibers based sensors exposed to 100 ppm ethanol (Eth), acetone (Ace), ammonia (Amm), methanol (Met), and toluene (Tol) at 350 °C.

It is well known that the response of a semiconductor metal oxide gas sensor is highly influenced by its operating temperature. Therefore, to begin with, ethanol vapor is used as the probe gas to perform gas-sensing tests at varying operating temperature to determine the optimum operating temperature. As shown in Fig. 4a, the sensing properties of two sensors to 100 ppm ethanol vapor are measured under different operating temperatures. Evidently, the output signal currents slightly increase with the increases of operating temperatures, indicating the decrease of resistance with temperature increasing. This temperature-dependent behavior of the samples is consistent with the normal semiconducting behavior. In addition, the relationship between the different operating temperatures and the corresponding sensor response is shown in insert figure of Fig. 4a. The sensitivity of the TiO_2_/Ag_0.35_V_2_O_5_ branched nanoheterostructures increases in relation to the operating temperature and reaches a maximum value of 31.8 at 350 °C. When the operating temperature increases beyond this value, the response value decreases due to the competition between adsorption and desorption of the chemisorbed gases. As for the pure TiO_2_ nanofibers, the sensitivity value increases with the operating temperature marginally and reaches 4.4 at 450 °C. By this token, the introducing of Ag_0.35_V_2_O_5_ can reduce the operating temperature evidently due to the heterojunction between TiO_2_ and Ag_0.35_V_2_O_5_ and the optimal operating temperature is determined to be 350 °C. Therefore, all sensing responses tests are further carried out at 350 °C for comparison.

The gas sensing performances of the TiO_2_/Ag_0.35_V_2_O_5_ branched nanoheterostructures and TiO_2_ nanofibers for ethanol vapor are circularly tested and plotted in Fig. 4b. The change in resistance of sensors is measured during a time period of 50 seconds at a temperature of 350 °C in all the cases. It shows that the resistance decreases after the introduction of ethanol gas and reaches a saturation stage. When the supply of ethanol gas is stopped, the resistance starts to increase again and returns to its original value. This typically shows an n-type semiconducting behavior. It can be inferred that the TiO_2_ nanofibers undergo a sensitivity value of about 3.5, whereas TiO_2_/Ag_0.35_V_2_O_5_ branched nanoheterostructures exhibit a sensitivity value of about 31.8, which is more than 9 times compared with the pure TiO_2_ nanofibers. For comparison, the gas sensing response of pure Ag_0.35_V_2_O_5_ nanofibers and TiO_2_/V_2_O_5_ fiber-like nanoheterostructures are also tested here (Fig. S4), where the response of Ag_0.35_V_2_O_5_ nanofibers is about 5.8, while the TiO_2_/V_2_O_5_ fiber-like nanoheterostructures exhibit improved gas sensing response of 24.8, indicating the hybridization of two semiconductors is much benefit to improve the gas sensing properties. Moreover, the better sensitive property of TiO_2_/Ag_0.35_V_2_O_5_ nanoheterostructures sensor compared with TiO_2_/V_2_O_5_ fiber-like nanoheterostructures sensor implies Ag_0.35_V_2_O_5_ is an outstanding choice for TiO_2_ modification to get enhanced ethanol sensitivity because of its excellent electrical conductivity^25^,^26^. In addition, the ethanol sensing properties of the TiO_2_/Ag_0.35_V_2_O_5_ nanoheterostructures and other n-n type TiO_2_-based nanoheterostructures published in recent literatures are compared and shown in Tab. S1. It can be seen that the TiO_2_/Ag_0.35_V_2_O_5_ nanoheterostructures sensor exhibits much higher ethanol gas sensing response compared with other competing nanoheterostructures^20^,^22^,^31^,^32^,^33^,^34^, this highly sensitive ethanol sensing property demonstrates high potential of TiO_2_/Ag_0.35_V_2_O_5_ nanoheterostructures for application in ethanol analysis. Furthermore, reproducibility, another important factor, is checked by repeating the response for ten times. It can be seen from Fig. 4b that both two samples exhibit outstanding reproducibility. The value for response and recovery times is also measured. The response time for TiO_2_/Ag_0.35_V_2_O_5_ branched nanoheterostructures and pure TiO_2_ nanofibers is calculated as 7 and 12 s, respectively, for 100 ppm of ethanol gas from the insert figure in Fig. 4b. Similarly, the recovery time of TiO_2_/Ag_0.35_V_2_O_5_ branched nanoheterostructures is calculated as 8 s, whereas pure TiO_2_ nanofibers have a very long recovery time of 13 s. The values indicate that the response and recovery times of TiO_2_/Ag_0.35_V_2_O_5_ branched nanoheterostructures are better than those of pure TiO_2_ nanofibers.

The dynamic response-recovery curves to different concentrations of ethanol vapor at 350 °C for TiO_2_/Ag_0.35_V_2_O_5_ branched nanoheterostructures and TiO_2_ nanofibers based sensors are plotted in Fig. 4c. It is clear to see that the TiO_2_/Ag_0.35_V_2_O_5_ branched nanoheterostructures based gas sensor presents excellent response-recovery characteristics to different concentrations of ethanol and the response amplitude increases with increasing the concentration from 20 ppm to 1000 ppm. For all five different ethanol gas concentrations of 20, 50, 100, 500, and 1000 ppm, the sensitivities of TiO_2_/Ag_0.35_V_2_O_5_ branched nanoheterostructures based sensor are 5.2, 15.0, 31.8, 39.9, and 42.6, respectively, while in the case of the pure TiO_2_ nanofibers, the sensitivities are 1.5, 2.4, 3.6, 3.8, and 3.8, respectively (inset in Fig. 4c). This confirms the improvement in sensing for TiO_2_/Ag_0.35_V_2_O_5_ branched nanoheterostructures. In summary, the sensor fabricated from TiO_2_/Ag_0.35_V_2_O_5_ branched nanoheterostructures exhibits higher sensitivity, shorter response time/recovery time, and broader detection range from 20 to 1000 ppm for ethanol sensing, compared with those obtained by pure TiO_2_ nanofibers.

To explore the selectivity of the TiO_2_/Ag_0.35_V_2_O_5_ branched nanoheterostructures sensor, other volatile organic pollutants (VOPs) including acetone, ammonia, methanol, and toluene are also measured under the same conditions and the result is shown in Fig. 4d. It is clear to see that the TiO_2_/Ag_0.35_V_2_O_5_ branched nanoheterostructures based sensor possesses a much higher response, not only to ethanol but also to ammonia and methanol, which are 31.8, 2.3, and 2.7, respectively, and are around 2–9 times compared with those of the pure TiO_2_ nanofibers sensor. Selectivity is another important aspect of the gas sensing performances. In fact, a sensor with good selectivity can be used to detect a specific target gas when it is exposed to a multicomponent gas environment. From Fig. 4d, it can be concluded that among all the five tested gases, the response of the TiO_2_/Ag_0.35_V_2_O_5_ branched nanoheterostructures based sensor to ethanol is the highest, and is 17.7, 13.8, 11.8, and 19.9 times higher than those to acetone, ammonia, methanol, and toluene, respectively, indicating its good selectivity in detecting ethanol.

### Gas sensing mechanism

**Figure 5:**
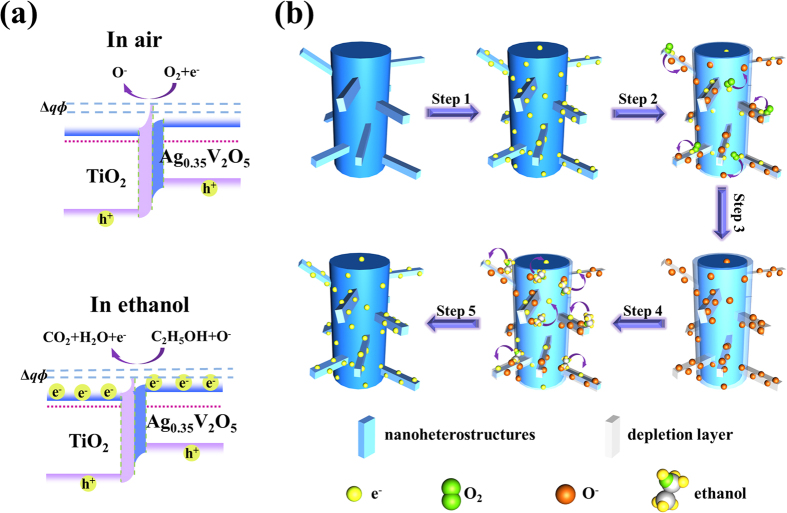
The scheme of the proposed gas sensing mechanism of the TiO_2_/Ag_0.35_V_2_O_5_ branched nanoheterostructures based sensor. (**a**) band structure model in air and in ethanol (E_V_: valence band; E_C_: conduction band; E_F_: Fermi level; qΦ: effective energy barrier of the heterojunction); (**b**) model of the TiO_2_/Ag_0.35_V_2_O_5_ branched nanoheterostructures based sensor exposed in air (step 1, step2, and step 3) and ethanol vapor (step 4 and step 5), respectively.

Based on the above results, the TiO_2_/Ag_0.35_V_2_O_5_ branched nanoheterostructures based sensor shows excellent sensing properties. Herein, we propose an analogous model for the TiO_2_/Ag_0.35_V_2_O_5_ branched nanoheterostructures based sensor (as shown in Fig. 5). First, a heterojunction can be formed at the interface between TiO_2_ and Ag_0.35_V_2_O_5_. Since the band gaps of TiO_2_ extrapolated from the UV-Vis spectrum using Tauc’s plot is close to the reported values in previous literature^20^,^22^ (Fig. S2), we employ the standard literature energy levels of TiO_2_ (conduction band of −3.9 eV, valance band of −7.1 eV, and Fermi level of −4.2 eV, vs. vacuum level, respectively) for the energy bands matching analysis here. In addition, Mott-Schottky testing is used to ascertain the conduction band of Ag_0.35_V_2_O_5_ here, the result shows that the conduction band of Ag_0.35_V_2_O_5_ is −5.12 eV vs. vacuum level (Fig. S5a). Considering the band gap of 2.1 eV extrapolated from the UV-Vis spectrum ((Fig. S2), the valance band of Ag_0.35_V_2_O_5_ should be −7.22 eV. Obviously, when the n-type semiconductor TiO_2_ and n-type semiconductor Ag_0.35_V_2_O_5_ contact with each other, an n-n type heterojunction can be formed. Because the Fermi level of TiO_2_ (−4.2 eV) is higher than that of Ag_0.35_V_2_O_5_ (−5.37 eV, Fig. S6), the electrons in the Ag_0.35_V_2_O_5_ will transfer to the TiO_2_ and result in a band bending between TiO_2_ and Ag_0.35_V_2_O_5_ interfaces, thus an energy barrier can be formed at the heterostructure interface (Fig. 5a). Second, oxygen species are adsorbed on the surface of the TiO_2_/Ag_0.35_V_2_O_5_ branched nanoheterostructures in the air condition, and then are ionized into oxygen ions (O^−^, O^2−^ and O_2_^−^) by capturing free electrons from the nanoheterostructures, thus leading to the formation of a thick depletion layer at the oxides surface and an increase of energy barrier height at the heterostructure interface (in air in Fig. 5a, step 1, 2, and 3 of Fig. 5b). Third, ethanol is a typical reductive gas, so when the sensor is exposed to ethanol gas, ethanol can react with the adsorbed oxygen species leading to the release of adsorbed electrons, the thinning of depletion layer at the oxides surface, and the decrease of energy barrier height at the heterostructure interface (in ethanol in Fig. 5a, step 4 and 5 of Fig. 5b). The mechanism can be explained by several chemical reactions, which are shown as follows:


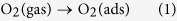



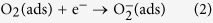



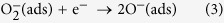



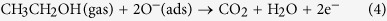


From the above reactions, it can be seen that the trapped electrons will be released to the TiO_2_/Ag_0.35_V_2_O_5_ branched nanoheterostructures after the supply of ethanol gas, thereby the carrier concentration and electron mobility on the sensor surface will be increased, then the depletion layer width and the energy barrier height will decrease and the resistance decrease accordingly. On the other hand, electrons on the conduction band will be captured by oxygen molecules adsorbed on the surface of the materials to form oxygen ions (O^−^, O^2−^ and O_2_^−^) after stopping ethanol gas supply, the depletion layer width and the energy barrier height will increase again, thus leading to an increase in resistance.

Therefore, a probable reason for the enhanced sensing properties of the TiO_2_/Ag_0.35_V_2_O_5_ is related to the extraordinary branched-nanofiber structures with branch diameter of about 20 nm and fiber diameter of about 160 nm according to the SEM results. On the one hand, the large BET surface area of the TiO_2_/Ag_0.35_V_2_O_5_ branched nanoheterostructures can be ascribed as one of most important factor for enhanced sensing performance. With the introducing of Ag_0.35_V_2_O_5_, the pure TiO_2_ nanofibers are transformed into branched-nanofibers, and the BET surface area of the nanoheterostructures is increased to 21.15 m^2^g^−1^, while for TiO_2_ nanofibers it is only 4.78 m^2^g^−1^ (Fig. 1f). This can provide more active sites for absorption of ethanol and reaction of ethanol with surface-adsorbed oxygen ions, thus the resistance decrease becomes more noticeable, and the gas sensing response is enhanced accordingly. On the other hand, electron exchange between the surface states and materials occurs within the surface layer, and the width of it is the order of the Debye length L_D_, which can be expressed by the following equation:


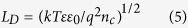


where k is the Boltzmann constant, T is the absolute temperature, ε is the static dielectric constant, ε_0_ is the permittivity of vacuum, q is the electrical charge of the carrier, and n_c_ is the carrier concentration. For the TiO_2_/Ag_0.35_V_2_O_5_ branched nanoheterostructures fabricated in this study, n_c_ of Ag_0.35_V_2_O_5_ extrapolated from the Mott-Schottky plot is about 9.6 × 10^18^ cm^−3^ (Fig. S5a), ε of Ag_0.35_V_2_O_5_ is measured to be 360 (see the methods for details). Accordingly, L_D_ is estimated to approximately 10 nm for Ag_0.35_V_2_O_5_ at 350 °C, this means that the depletion layer of the Ag_0.35_V_2_O_5_ is equivalent to the semidiameter of the branches, thus electrons in the Ag_0.35_V_2_O_5_ branches can be outright depleted by surface adsorbed O_2_ molecules^35^ (as shown in the fourth figure in Fig. 5b). Whereas for the TiO_2_/Ag_0.35_V_2_O_5_ backbone or TiO_2_ nanofibers, the depletion layer of 3–30 nm for metal oxides^36^ is far away from the semidiameter of the nanofibers (the fourth figure in Fig. 5b). The entire depletion of the carriers in Ag_0.35_V_2_O_5_ branches can induce much evident change in resistance, and consequently the resistance decrease of the TiO_2_/Ag_0.35_V_2_O_5_ branched nanoheterostructures is more obvious than that of pure TiO_2_ nanofibers after exposure in ethanol gas.

Another probable explanation for the enhancement of the TiO_2_/Ag_0.35_V_2_O_5_ gas sensor is the formation of n-n type heterojunctions between TiO_2_ and Ag_0.35_V_2_O_5_. Since the work function of TiO_2_ (−4.2 eV) is larger than that of Ag_0.35_V_2_O_5_ (−5.37 eV), the electrons in the TiO_2_ will transfer to the Ag_0.35_V_2_O_5_, thus resulting in an energy barrier and an additional depletion layer at the interfaces. Compared with pure TiO_2_ or Ag_0.35_V_2_O_5_, the conduction channel of TiO_2_/Ag_0.35_V_2_O_5_ nanoheterostructures is much influenced by the energy barrier. The resistance of the heterojunctions can be expressed by the following equation:


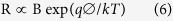


where B is a constant, k is the Boltzmann constant, T is the absolute temperature and qΦ is effective energy barrier at the heterojunction. For air condition, the effective energy barrier (qΦ) increases because the free electrons are captured by oxygen species to ionize into oxygen ions (O^−^, O^2−^ and O_2_^−^) (as shown in the first figure in Fig. 5a). After exposure in ethanol gas, ethanol can react with the adsorbed oxygen species and lead to the release of adsorbed electrons, thus leading to the decrease of the energy barrier (the second figure in Fig. 5a). It is obvious that R_a_/R_g_ is in direct proportion to the value of exp(ΔqΦ), so the remarkable changes of energy barrier of the heterojunctions can induce great change in the conductivity and improvement of the gas-sensing performance^32^, which can be entitled as synergistic effect. Additionally, the heterojunctions can also be used for additional active sites, leading to an improvement in the sensing performances^37^,^31^. What is more, the TiO_2_/Ag_0.35_V_2_O_5_ branched nanoheterostructures act as a more efficient catalyst than pure TiO_2_ nanofibers^38^, which can promote the sensing reaction between the reductive VOPs and adsorbed oxygen species^34^.

From all the above, the high performance of the TiO_2_/Ag_0.35_V_2_O_5_ branched nanoheterostructures gas sensor for ethanol can be ascribed to the following two factors. First, the enhancement in gas sensing is believed to be related to the novel branched-nanofiber structure, which display larger BET surface area and completely electrons depletion for nanobranches compared with the pure TiO_2_ nanofibers. Secondly, the synergistic effect, additional active sites, and efficient catalytic capability induced by the effective heterojunctions between TiO_2_ and Ag_0.35_V_2_O_5_ also contribute to the gas sensing enhancement.

In conclusion, we have demonstrated a high ethanol sensitivity and selectivity for TiO_2_/Ag_0.35_V_2_O_5_ branched nanoheterostructures based gas sensor. Compared with the pure TiO_2_ nanofibers sensor, the gas response of the TiO_2_/Ag_0.35_V_2_O_5_ branched nanoheterostructures sensor is dramatically enhanced by 9.1 times at 350 °C for ethanol (R_a_/R_g_ = 31.8 at 100 ppm), In addition, the TiO_2_/Ag_0.35_V_2_O_5_ branched nanoheterostructures sensor exhibits a faster response/recovery time (7/8 s at 100 ppm), and a better selectivity characteristic toward ethanol. The gas sensing enhancement arises for the extraordinary branched structures and TiO_2_/Ag_0.35_V_2_O_5_ heterojunctions of the TiO_2_/Ag_0.35_V_2_O_5_ branched nanoheterostructures. These results reveal the mechanism of chemical sensing performance enhancement for branched nanoheterostructures. The paper also outlines an approach to further optimize the nanostructure based chemical sensors.

## Methods

### Fabrication of TiO_2_/Ag_0.35_V_2_O_5_ branched nanoheterostructures

The TiO_2_/Ag_0.35_V_2_O_5_ branched nanoheterostructures were prepared by an electrospinning process followed by an annealing treatment^39^. First, 0.50 g tetrabutyltitanate (TBT) and 0.20 g polyvinylpyrrolidone (PVP) were dissolved in a mixture of 1.50 ml ethanol and 1.20 ml acetic acid, and stirred for 20 min to give PVP/TBT composite. Then, 0.60 g PVP, 0.20 g VO(acac)_2_, and 0.035 g Ag(NO)_3_ were added into 3.70 g dimethylacetamide (DMAC), after stirring for 20 min, the resulting solution was mixed with the PVP/TBT composite prepared in the first step and stirred for 1 h to prepare PVP/TBT/Ag(NO)_3_/VO(acac)_2_ composite. Next, the PVP/TBT and PVP/TBT/Ag(NO)_3_/VO(acac)_2_ composites were electrospun and then annealed at 450 °C in ambient air for 1 h to remove the PVP support, crystallize TiO_2_ and Ag_0.35_V_2_O_5_, and finally resulted in TiO_2_ nanofibers and TiO_2_/Ag_0.35_V_2_O_5_ branched nanoheterostructures (Ti/V molar ratio is 1), respectively. In a typical electrospinning process, the spinneret had an inner diameter of 0.4 mm. A distance of 15 cm and DC voltage of 15 kV were maintained between the tip of the spinneret and the collector. Additionally, Ag_0.35_V_2_O_5_ nanofibers and TiO_2_/V_2_O_5_ fiber-like nanoheterostructures for comparison were prepared by the same electrospinning process using PVP/Ag(NO)_3_/VO(acac)_2_ composite and PVP/TBT/VO(acac)_2_ composites, respectively. TiO_2_/Ag_0.35_V_2_O_5_ and Ag_0.35_V_2_O_5_ films are prepared by electrospinning the nanofibers onto FTO glass substrates for Mott-Schottky testing.

### Characterization and gas sensing measurements

The morphologies of the samples were characterized by field emission scanning electron microscopy (FESEM, Ultra 55) and transmission electron microscopy (TEM, Libra 200FE). X-ray diffraction (XRD, CuKα, λ = 1.5406 Å, X’Pert PRO) and high-resolution TEM (HRTEM) were employed to characterize the crystal structure and elemental analysis of the samples. Nitrogen adsorption-desorption isotherms (ASAP 2020 nitrogen adsorption apparatus) was employed to measure the Brunauer-Emmett-Teller (BET) specific surface areas of the samples. The chemical composition was determined by X-ray photoelectron spectroscopy (XPS), and the measurements were performed in a VG Scientific ESCALAB 210 spectrometer equipped with Mg anode and a source power of 300 W. All binding energies were calibrated to the C 1s peak at 284.6 eV. The UV-Vis absorption spectra were recorded using a UV-3150 spectrophotometer to evaluate the absorption properties. Mott-Schottky testing was performed at an electrochemistry workstation (RST5200) to obtain the semiconductor type, carrier concentration, and conduction band energy of the samples. The measurements were performed in a three-electrode cell with 0.2 M Na_2_SO_4_ (PH = 6.5) at a frequency of 1 kHz and scan rate of 10 mV/s, where Pt wire was used as the counter electrode and Ag/AgCl electrode was used as the reference electrode. The potential was measured against an Ag/AgCl reference electrode and converted to NHE potentials using *E* (NHE) = *E* (Ag/AgCl) + (0.059 × pH) + 0.197 V. The Fermi energy level of the Ag_0.35_V_2_O_5_ sample was measured by the Kelvin probe force microscopy (KPFM) using the SII E-Sweep SPM system in air condition at room temperature across the Au/Ag_0.35_V_2_O_5_ border, which was formed at the surface of Ag_0.35_V_2_O_5_ by depositing a stripe of Au film. Furthermore, the static dielectric constant was tested using an Agilent 4294A Precision LCE Meter (Agilent Technologies Inc.) at the frequency of 10 MHz.

The preparation of the gas sensor was similar to that depicted in previous literature^40^. The sensor device was prepared by dispersing the TiO_2_/Ag_0.35_V_2_O_5_ branched nanoheterostructures into ethanol to form a paste and coated onto the outside surface of an alumina tube which was printed a pair of Au electrodes previously. Then, the sensor devices were dried at 150 °C for 3 h in ambient air to form sensor film. Finally, a Ni-Cr alloy wire was inserted into the alumina tube and employed as a heater, the operating temperatures were controlled by adjusting the heating power of the alloy. The gas-sensing properties were measured under a steady-state condition by using a high precision sensor testing system (WS-30A). The device was examined at 50% relative humidity in the temperature range of 250–450 °C at various concentrations of ethanol (20–1000 ppm). The sensor response was defined as S = R_a_/R_g_, where R_a_ is the resistance in air and R_g_ is the resistance in the probe gas. The response time was defined as the time needed for the variation in electrical resistance to reach 90% of the equilibrium value after injecting ethanol, and the recovery time was defined as the time needed for the sensor to return to 90% above the original resistance in air after removing the ethanol.

## Additional Information

**How to cite this article**: Wang, Y. *et al*. A novel ethanol gas sensor based on TiO_2_/Ag_0.35_V_2_O_5_ branched nanoheterostructures. *Sci. Rep.* **6**, 33092; doi: 10.1038/srep33092 (2016).

## Supplementary Material

Supplementary Information
