# Supplementary material for: A novel ethanol gas sensor based on TiO2/Ag0.35V2O5 branched nanoheterostructures
Source: Sci Rep. 2016 Sep 12;6:33092. doi: 10.1038/srep33092 (PMC5018879; doi:10.1038/srep33092)
Supplement: Supplementary Information [file srep33092-s1.pdf]

Supplementary Materials:

A novel ethanol gas sensor based on $TiO_2/Ag_{0.35}V_2O_5$ branched nanoheterostructures

Yuan Wang*, Lixin Liu*, Chuanmin Meng, Yun Zhou, Zhao Gao, Xuhai Li, Xiuxia Cao, Liang Xu, Wenjun Zhu,

National Key Laboratory of Shock Wave and Detonation Physics, Institute of Fluid Physics, China Academy of Engineering Physics, P. O. Box 919-111, Mianyang, Sichuan 621900, People's Republic of China.

*Corresponding authors: YuanWang: E-mail: wangyuan0000@gmail.com, Lixin Liu: E-mail: liulix00@gmail.com; Fax: +86 0816 2485139; Tel: +86 0816 2485139

1. Photographs and absorption spectra

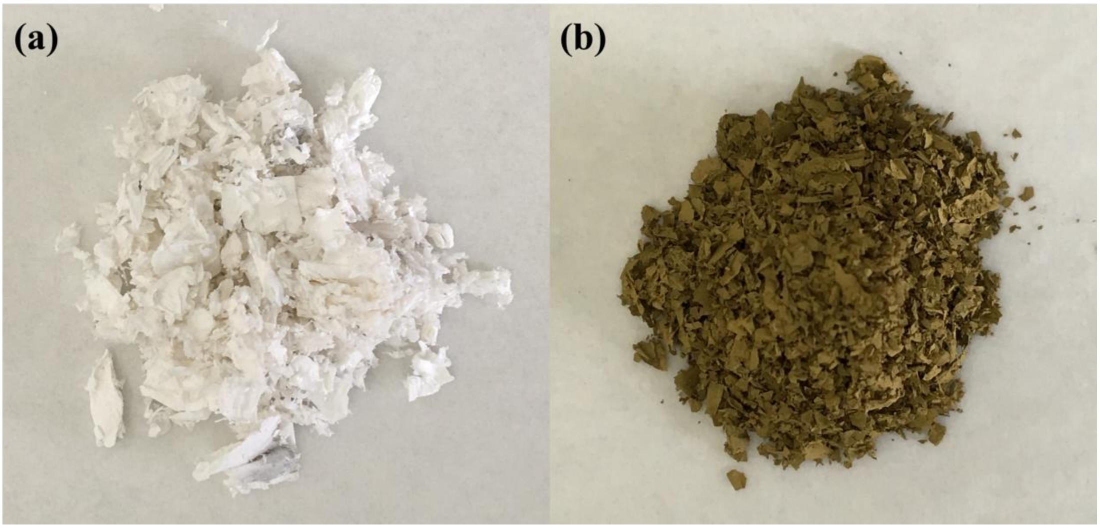

Figure S1. Photographs for comparing white TiO_2 nanofibers and brown $TiO_2/Ag_{0.35}V_2O_5$ nanoheterostructures.

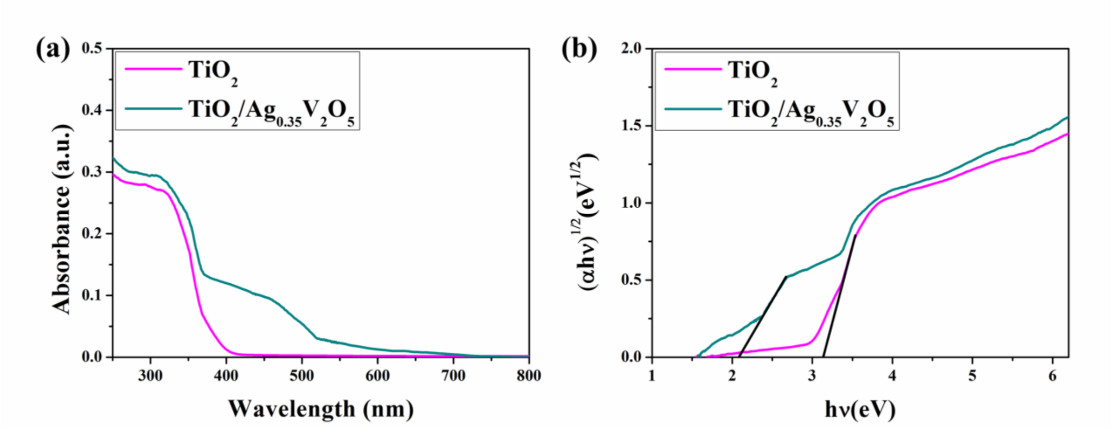

Figure S2. (a) UV-Vis absorption spectra of TiO_2 nanofibers and $TiO_2/Ag_{0.35}V_2O_5$ nanoheterostructures. (b) Tauc's plots for the TiO_2 and $Ag_{0.35}V_2O_5$ in TiO_2 nanofibers and $TiO_2/Ag_{0.35}V_2O_5$ nanoheterostructures, respectively.

The color of the two samples is very different, as can be clearly seen in Fig. S1, the color of TiO_2 nanofibers is white, while the $TiO_2/Ag_{0.35}V_2O_5$ branched nanoheterostructures turn to brown, indicating $Ag_{0.35}V_2O_5$ are successfully introduced to TiO_2 host.

UV-Vis absorption curves of the $TiO_2/Ag_{0.35}V_2O_5$ branched nanoheterostructures and pure TiO_2 nanofibers are measured and shown in Fig. S2a. It is clear that the maximum absorbance peak of the pure TiO_2 nanofibers occurs at ultraviolet range and almost has no absorbance for visible light due to its large band gap energy (3.2 eV). When coupling with the $Ag_{0.35}V_2O_5$, the $TiO_2/Ag_{0.35}V_2O_5$ nanoheterostructures show an additional broad absorption band from 400 nm to 700 nm, indicating the effective photo-absorption ability for the $Ag_{0.35}V_2O_5$ and heterostructures, in consistent with the dramatic color change of the samples (Fig. S1). Meanwhile, the band gaps of the TiO_2 and $Ag_{0.35}V_2O_5$ extrapolated from the UV-Vis spectrum using Tauc's plot are about 3.2

eV and 2.1 eV, respectively, it can be seen that the band gap energy of TiO_2 extrapolated here is close to the reported values in previous literature[1] (Fig. S2b).

2. XPS spectra

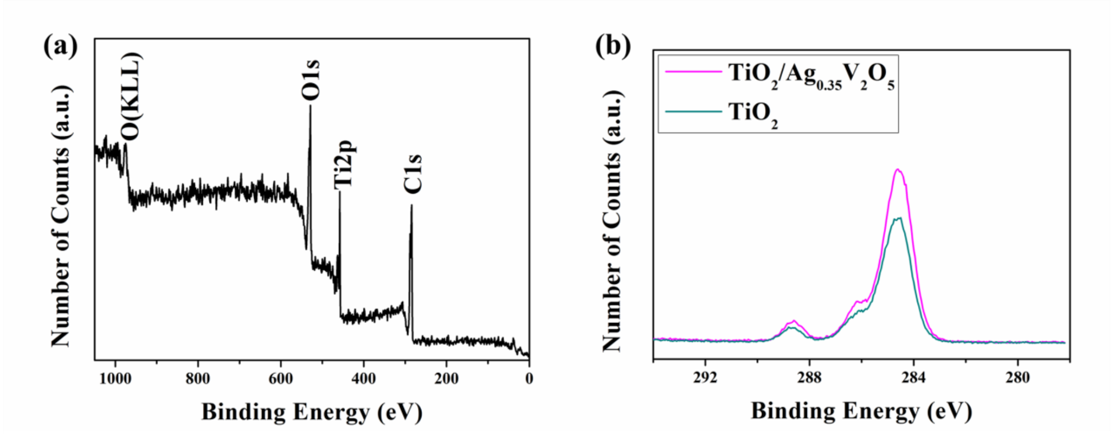

Figure S3. XPS spectra: (a) survey spectrum of TiO_2 nanofibers, (b) C1s core-level spectra of TiO_2 nanofibers and $TiO_2/Ag_{0.35}V_2O_5$ branched nanoheterostructures, illustrating the binding energies are calibrated to the C 1s peak at 284.6 eV.

The whole survey for all elements detection of the TiO_2 nanofibers is presented in Fig. S3a illustrating the successful synthesis of the TiO_2 nanofibers. The C 1s core-level spectra of TiO_2 nanofibers and $TiO_2/Ag_{0.35}V_2O_5$ branched nanoheterostructures are shown in Fig. S3b, where the peak at 284.6 eV illustrates all the binding energies of the two samples are calibrated to the C 1s peak at 284.6 eV.

3. Gas sensing properties

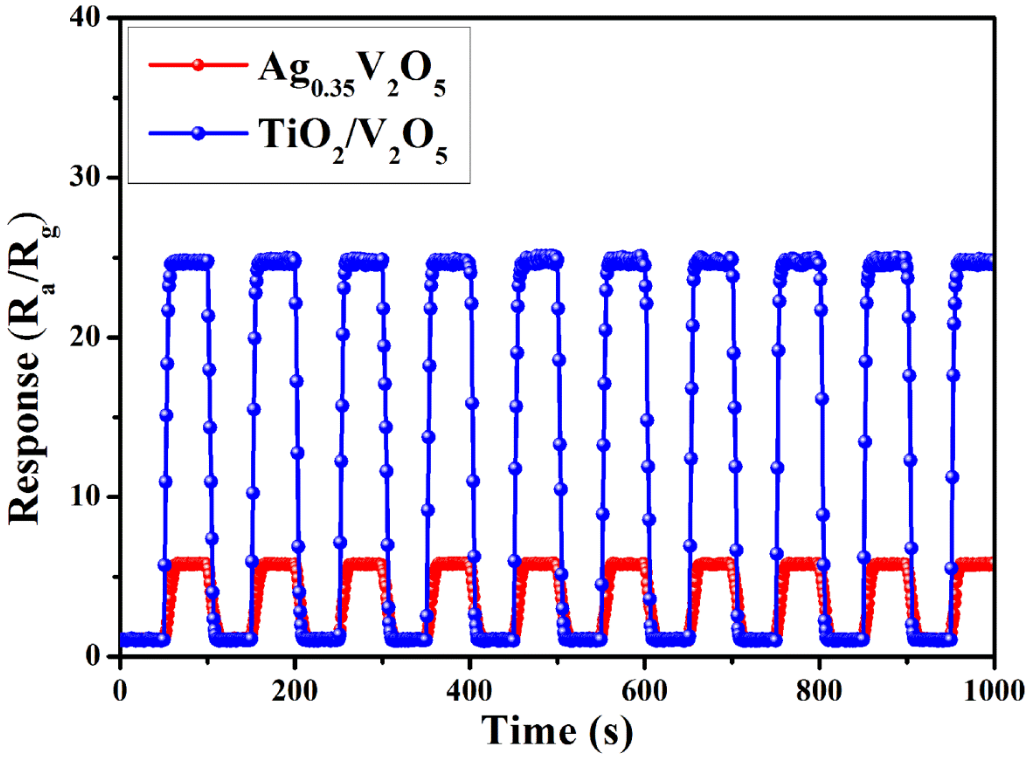

Figure S4. Gas sensing response of $Ag_{0.35}V_2O_5$ nanofibers and TiO_2/V_2O_5 fiber-like nanoheterostructures exposed to 100 ppm successive ethanol vapors (10 cycles) at 350 ^oC.

The gas sensing response of $Ag_{0.35}V_2O_5$ nanofibers and TiO_2/V_2O_5 nanoheterostructures are tested at 100 ppm successive ethanol vapors (10 cycles) at 350 ^oC for comparison. As shown in Fig. S4, the ethanol sensing response of $Ag_{0.35}V_2O_5$ nanofibers is about 5.8, higher than that of pure TiO_2 nanofibers while much lower than that of $TiO_2/Ag_{0.35}V_2O_5$ nanoheterostructures, indicating the hybridization of the two semiconductors is benefit to the enhancement of gas sensing properties. As for the TiO_2/V_2O_5 fiber-like nanoheterostructures, the sensor exhibits improved gas sensing response of 24.8 than pure TiO_2 nanofibers, but still inferiors to $TiO_2/Ag_{0.35}V_2O_5$ nanoheterostructures, this may derive from the poor electrical

conductivity of V_2O_5 compared with $Ag_{0.35}V_2O_5$[2].

Table S1. Comparison of the responses, response times, and recovery times of the $TiO_2/Ag_{0.35}V_2O_5$ nanoheterostructures sensor to ethanol vapor with those of other n-n type TiO_2-based nanoheterostructures sensors.

Nanomaterials	Ethanol concentration (ppm)	Work temperature (°C)	Response (Ra/Rg)	Response time (s)	Recovery time (s)	Reference
$TiO_2/Ag_{0.35}V_2O_5$ nanoheterostructures	100	350	31.8	7	8	This work
TiO_2/V_2O_5 nanoheterostructures	100	350	24.8	6	7	This work
TiO_2/ZnO heterojunctions	100	320	13.2	5-10	5-10	3
TiO_2/ZnO nanofibers	100	280	15.7	5	3	4
TiO_2/SnO_2 hybrid oxides	100	320	10	-	-	5
TiO_2/SnO_2 nanobelts	100	250	25.7	-	-	6
TiO_2/SnO_2 core shell nanocomposites	1000	230	12.7	<50	<50	7
TiO_2/Fe_2O_3 tube-like nanostructures	100	270	8.2	-	-	8
$TiO_2/\alpha\text{-}Fe_2O_3$ nanoheterostructures	100	370	14.2	5-7	4-6	9

Tab. S1 is the summary of the recent publications related to the n-n type TiO_2-based nanoheterostructures as well as $TiO_2/Ag_{0.35}V_2O_5$ nanoheterostructures for ethanol sensing layers. It is obvious that the $TiO_2/Ag_{0.35}V_2O_5$ nanoheterostructures sensor exhibits much higher ethanol gas sensing response compared with other competing nanoheterostructures when measured at the same ethanol concentration [3-6, 8, 9].

Additionally, for different ethanol concentration, the response of $TiO_2/Ag_{0.35}V_2O_5$ nanoheterostructures sensor to 100 ppm ethanol is larger than that of TiO_2/SnO_2 core shell nanocomposites sensor to 1000 ppm ethanol[7]. This highly sensitive ethanol sensing property demonstrates high potential of $TiO_2/Ag_{0.35}V_2O_5$ nanoheterostructures for application in ethanol analysis.

4. Energy levels

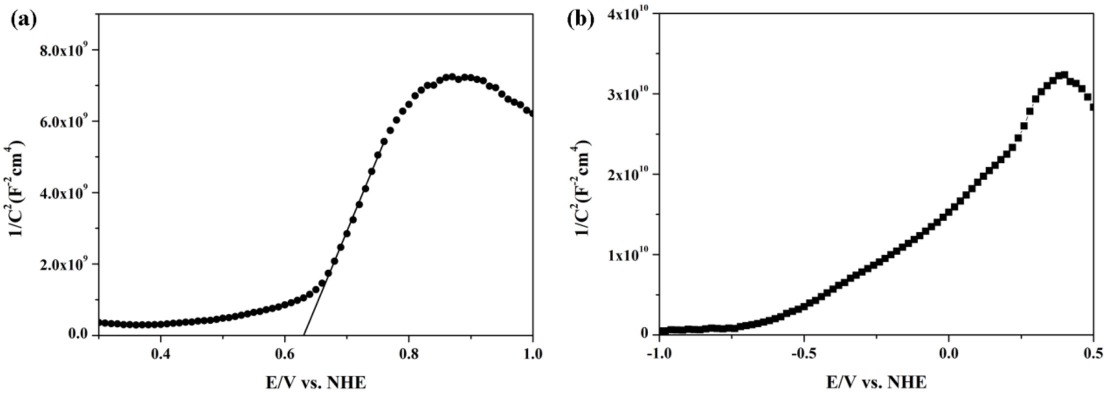

Figure S5 Mott-Schottky plots for (a) $Ag_{0.35}V_2O_5$ nanofibers and (b) $TiO_2/Ag_{0.35}V_2O_5$ nanoheterostructures.

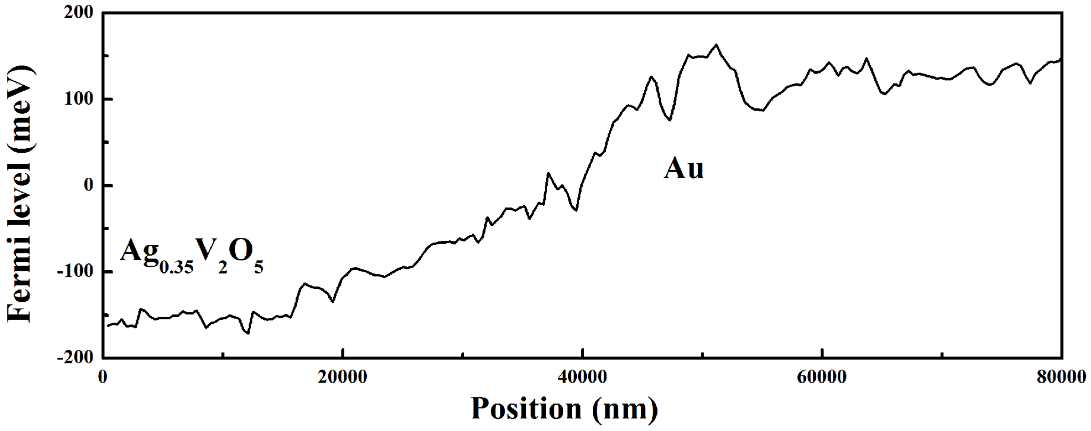

Figure S6 Fermi energy level difference between the Au and $Ag_{0.35}V_2O_5$ area at the

$Au/Ag_{0.35}V_2O_5$ border.

To obtain the electrochemical properties of the $Ag_{0.35}V_2O_5$, we perform electrochemical impedance measurements on the $Ag_{0.35}V_2O_5$ film prepared on FTO glass substrate. The conducting type, carrier concentration and band potential of the $Ag_{0.35}V_2O$ can be extrapolated using the Mott–Schottky equation[10]:

$$1/C^2 = (2/q\varepsilon\varepsilon_0 N_c)[(V - V_f) - kT/e_0] \quad (1)$$

where q is the electron charge, ε is the dielectric constant of $Ag_{0.35}V_2O_5$, ε_0 is the permittivity of vacuum, N_c is the carrier concentration, V is the electrode potential, V_f is the flat band potential, T is the temperature and k is Boltzmann's constant. As shown in Fig. S5, the positive slope indicates an n-type semiconductor of the $Ag_{0.35}V_2O_5$ and the $TiO_2/Ag_{0.35}V_2O_5$. By extrapolating the X intercept, the V_f of the $Ag_{0.35}V_2O_5$ is determined to be 0.62 V vs. NHE (Fig. S5a). It is well known that the flatband potential is considered to be located just under the conduction band for n-type semiconductors, hence the conduction band of the $Ag_{0.35}V_2O_5$ is estimated to be 0.62 eV vs. NHE or -5.12 eV vs. vacuum level. Based upon the band gap of 2.1 eV extrapolated from the UV-Vis spectrum (Fig. S2b), the valance band of the $Ag_{0.35}V_2O_5$ is inferred to be -7.22 eV vs. vacuum level. Furthermore, Fermi energy level of the $Ag_{0.35}V_2O_5$ sample is measured by the Kelvin probe force microscopy (KPFM), the Fermi energy level difference between Au and $Ag_{0.35}V_2O_5$ area is recorded and shown in Fig. S6. It is found that the Fermi energy level of the $Ag_{0.35}V_2O_5$ lies ~276 meV below that of Au, considering the work function of 5.1 eV for Au, the work function of the $Ag_{0.35}V_2O_5$ is determined to be 5.37 eV.

The carrier concentration of the $Ag_{0.35}V_2O_5$ can also be calculated from the slope of the Mott Schottky plot[10]:

$$N_c = (2/q\varepsilon\varepsilon_0)[d(1/C^2)dV]^{-1} \qquad (2)$$

With ε of 360 tested at the frequency of 10 MHz, the carrier concentration is calculated to be $9.6x10^{18}$ cm^{-3}.

References

1 Zhu, C. L. et al. Fe_2O_3/TiO_2 tube-like nanostructures: synthesis, structural transformation and the enhanced sensing properties. *ACS Appl. Mater. Interfaces* **4**, 665-671 (2012).

2 Xiong, C., Aliev, A. E., Gnade, B. & Balkus Jr, K. J. Fabrication of silver vanadium oxide and V_2O_5 nanowires for electrochromics. *ACS nano* **2**, 293-301 (2008).

3 Deng, J. et al. Facile synthesis and enhanced ethanol sensing properties of the brush-like ZnO-TiO_2 heterojunctions nanofibers. *Sens. Actuat. B: Chem.***184,** 21- 26 (2013).

4 Lou, Z. et al. A class of hierarchical nanostructures: ZnO surfacefunctionalized TiO_2 with enhanced sensing properties. *RSC Adv.* **3**, 3131-3136 (2013).

5 Zeng, W., Liua, T. and Wang Z. Enhanced gas sensing properties by SnO_2 nanosphere functionalized TiO_2 nanobelts. *J. Mater. Chem.* **22**, 3544-3548 (2012).

6 Chen, G. et al. High-energy faceted SnO_2-coated TiO_2 nanobelt heterostructure

for near-ambient temperature-responsive ethanol sensor. *ACS Appl. Mater. Interfaces* **7**, 24950-24956 (2015).

7 Vaezi, M. R., Shendy, S. K. and Ebadzadeh T. Synthesis of TiO_2/SnO_2 core shell nanocomposite by chemical route and its gas sensing properties. *Indian J. Phys.* **86**, 9-13 (2012).

8 Zhu, C. L. et al. Fe_2O_3/TiO_2 tube-like nanostructures: synthesis, structural transformation and the enhanced sensing properties. *ACS Appl. Mater. Interfaces* **4**, 665-671 (2012).

9 Wang, Y. et al. Brookite TiO_2 decorated a-Fe_2O_3 nanoheterostructures with rod morphologies for gas sensor application. *J. Mater. Chem. A* **2**, 7935-7943 (2014).

10 Yang, X. Y. et al. Nitrogen-doped ZnO nanowire arrays for photoelectrochemical water splitting. *Nano Lett.* **9,** 2331–2336 (2009).
